# Cognitive-linguistic profiles of Chinese typical-functioning adolescent dyslexics and high-functioning dyslexics

**DOI:** 10.1007/s11881-018-0165-y

**Published:** 2018-08-17

**Authors:** Kevin Kien Hoa Chung, Jason C. M. Lo, Catherine McBride

**Affiliations:** 1 Department of Early Childhood Education, Department of Special Education and Counselling, and Centre for Child and Family Science, The Education University of Hong Kong, Hong Kong, China; 2 Department of Psychology, The Chinese University of Hong Kong, Hong Kong, China

**Keywords:** Adolescents, Chinese language, Dyslexia, Multiple deficits, Rapid naming

## Abstract

Dyslexia is a developmental disability affecting the acquisition of reading and writing skills, and its developmental nature makes longitudinal research of great importance. This study therefore investigated the cognitive-linguistic profiles of the typical-functioning dyslexics and high-functioning dyslexics with longitudinal cohorts of Chinese-speaking adolescents diagnosed with childhood dyslexia. These two dyslexic groups of fifty students (with 25 typical-functioning dyslexics) were assessed in Grade 2 (Time 1) and in Grade 8 (Time 2), whereas 25 typically developing controls were assessed at Time 2. Students were administered measures of phonological awareness, morphological skills, visual-orthographic knowledge, rapid naming, verbal working memory, and literacy skills. Results showed that, at Time 2, both dyslexic groups performed less well than the control group on most of the measures. Deficits in rapid naming were particularly salient in both dyslexic groups. Comparing the two dyslexic groups, the typical-functioning dyslexics had more multiple deficits than the high-functioning dyslexics. Findings highlight the importance of rapid naming deficits as potential universal causes of dyslexia and the utility of targeting visual-orthographic knowledge and morphological skills in supporting the development of dyslexic adolescents.

Developmental dyslexia (hereafter, dyslexia), characterized by difficulties with word decoding skills, presents in approximately 4 to 10% of individuals and can affect word reading accuracy and fluency as well as spelling in spite of normal intelligence and adequate formal education (Chung, 2017; Chung & Ho, 2010; Gabrieli, 2009; Vellutino, Fletcher, Snowling, & Scanlon, 2004). Although dyslexia is a lifelong disability, the severity and manifestations of the condition may change over time. Due to its developmental nature, the cognitive-linguistic characteristics of dyslexia arising in childhood may or may not change across time or be resolved with cognitive maturation and development. Moreover, the quantity and quality of problems may differ among students with dyslexia from childhood through adolescence. With proper intervention and school support, a variety of deficits in cognitive-linguistic skills and literacy skills can be improved to varying degrees over time (e.g. Birch & Chase, 2004; Pammer, 2014). Indeed, many students diagnosed as dyslexic in childhood can achieve functional or normal literacy skills to enable academic study at higher levels (Pennington et al., 1986) and are sometimes referred to as high-functioning dyslexics. However, the literacy skills of the majority of typical-functioning dyslexics remain low, thus making academic study very difficult (Birch & Chase, 2004; Hoeft et al., 2011).

Although high-functioning dyslexics have been found among those whose language is alphabetic such as English, relatively little is known about the incidence in non-alphabetic languages like Chinese about how Chinese students with childhood dyslexia go on to develop those cognitive-linguistic skills and literacy skills in secondary school grades. As proposed by Bishop and Snowling (2004), the characteristics of a given language may affect the manifestation of cognitive-linguistic deficits linked to literacy difficulties. Thus, in the present study, we aimed to address the issue of what happens to Chinese children with dyslexia when they become adolescents, as compared to typically developing adolescents. Particularly, this study examined the cognitive-linguistic profiles of students with high-functioning and typical-functioning dyslexia through primary to secondary school in Hong Kong. Many studies on dyslexia in alphabetic languages such as English have shown that the major causes of reading and writing problems lie with deficits in the phonological domain, specifically in the access to phonological representations and phonological processing skills (Snowling, 2000; Ramus & Szenkovits, 2008). These deficits make it difficult for children with dyslexia to read and spell unfamiliar words as they affect the skills of analyzing and manipulating sound structures of words and linking letter sound correspondence. Due to the close relationships between phonology and orthography in English, phonological sensitivity is particularly important in learning to read and write and this in turn makes it the key indicator of reading and writing difficulties.

Also importantly, deficits in rapid naming (RAN), i.e. measured time to articulate colors, objects, digits, or letters, have been considered another major core of deficit in dyslexia (Norton & Wolf, 2012; Wolf & Bowers, 1999) because rapid naming deficits tend to signify generally weak phonological decoding, less automatic processes of extraction and induction of visual and orthographic patterns, and slow general processing speed in the mental lexicon (Bowers & Wolf, 1993; Wolf & Bowers, 1999). The extant evidence also suggests that deficits in rapid naming, apart from phonological difficulties, many children with dyslexia experience persist into adulthood (Bruck, 1992; Felton, Naylor, & Wood, 1990). Furthermore, other cognitive-linguistic causes of dyslexia, for example, orthographic and morphological processing and verbal working memory may be independent but perhaps may stem from deficits in phonological processing and rapid naming (e.g. Burt, 2006; Corcos & Willows, 1989; Jeffries & Everatt, 2004; Law, Wouters, & Ghesquière, 2015). These deficits generally impact children’s ability to encode print words, manipulate the structure of words, and analyze the meaning of morphologically complex words in verbal working memory. Few studies have looked at what underlies dyslexia in languages like Chinese that have a distinctively different writing system, but interest in Chinese individuals with diagnosed childhood dyslexia is increasing. The discussion of the cognitive-linguistic causes of dyslexia in Chinese is preceded by a brief description of the features of the Chinese orthography.

Chinese varies from the English writing system in the relations among orthography, phonology, morphology, and semantics. Unlike English, Chinese is a morphosyllabic writing system in which the basic graphic unit is a character representing both a syllable and morpheme (DeFrancis, 1989; Mattingly, 1984). The correspondence between morpheme and syllable is almost one to one across Chinese dialects and scripts. In Chinese, characters are made up of different stroke patterns that provide rich visual-spatial properties (e.g., <一>, <丨>, <ヽ>, <宀>, <广>), (Chen & Kao, 2002; Gao & Kao, 2002). These stroke patterns are the building components of radicals that are combined to form Chinese characters and thereby provide a perceptual aspect of orthographic processing (Shen & Bear, 2000). Traditional characters, which are regularly used in Hong Kong, require even more stroke patterns than do the simplified characters used in mainland China. For example, the character <體> (/tai2/, meaning ‘body’), has 23 strokes in traditional form, whereas its simplified form <体> consists of 7. Chinese characters can be formed as one-radical simple characters or multi-radical compound characters (Huang, 2005; Lin, 2006).

More than 80% of Chinese characters are composed of ideophonetic compounds with each component (or radical) comprising a semantic and a phonetic radical (Kang, 1993). For example, a Chinese character, <楓> (/fung1/, meaning ‘maple tree’), consists of two components with the left representing the semantic radical <木> meaning tree and the right signifying a phonetic radical which is pronounced as <風>, /fung1/ in Cantonese dialect, which is mainly spoken in south-eastern parts of China, including Hong Kong. Most semantic radicals inhabit the left or top position within left-right such as <烤> (/haau1/, ‘grill’) or top-bottom <花> (/faa1/, ‘flower’) structure. However, phonetic radicals are not always reliable predictions of the pronunciation of a character. Only 40% of the characters can be directly decoded from their respective phonetic radical by using orthography-phonology correspondence rules (Shu, Chen, Anderson, Wu, & Xuan, 2003). Semantic radicals generally appear to be more reliable than the phonetic radicals given that different degrees of semantic and phonological regularity/consistency exist in Chinese characters. There are over 800 phonetic radicals (DeFrancis, 1984) and around 200 semantic radicals (Feldman & Siok, 1999) with different degrees of positional, semantic, and phonological regularities for radicals; the orthographic rules in Chinese are visually compact and relatively complicated. Since learning of Chinese characters tends to rely heavily on the relatively arbitrary associations between print and sound, readers’ knowledge about internal structures and positions of radicals within characters plays an important role in reading and writing Chinese.

Another unique feature of Chinese that differs from English relates to aspects of morphological processing. One aspect of morphological awareness in Chinese involves the number of homophones and homographs. Chinese has a great many syllables that have more than one homophone, and each syllable provides different meaning (Packard, 2000; Zhou, Zhuang, & Yu, 2002). For example, the syllable /hung4/ has the different meanings of ‘red’, ‘bear’, ‘male’ and ‘flood.’ Furthermore, a morpheme (or character) can be combined with two or more morphemes to create compound words in Chinese (Packard, 2000). To illustrate, the morpheme <火> (fire) can be compounded with other characters to form several new words, e.g., <火災> (fire hazard), <火爐> (fire furnace), and <火石> (firestone). These words share the fire morpheme in common and are morphologically related as a consequence. Thus, morphological skills, the ability to manipulate morphemes and employ rules of word formation, have significant influence on reading and writing (e.g., Kalindi & Chung, 2018; McBride-Chang, Lam, Lam, Doo, Wong, & Chow, 2008; Shu et al., 2006). Because numerous complex rules of orthographic regularities, and an abundance of homophones and homographs, are required to be stored in and retrieved from the lexicon in Chinese, rapid naming, which reflects the speed of access to the lexicon, and verbal working memory are also particularly important for reading and writing acquisition and difficulties.

Given the different linguistic features of Chinese as mentioned above, the cognitive-linguistic manifestations of Chinese individuals with dyslexia may be different from those of dyslexic individuals in English. For example, studies carried out in Hong Kong have found that Chinese children with dyslexia often display deficits in multiple cognitive-linguistic domains, predominantly rapid naming, visual-orthographic knowledge, and verbal working memory (Ho et al., 2002, 2004). These children, who have multiple cognitive-linguistic deficits, tend to manifest more severe difficulties in reading and writing as compared to those children with deficits in a single domain. Furthermore, phonological deficits tend to be relatively less common in Chinese children with dyslexia compared to their English counterparts (Ho et al., 2004). It is noteworthy that Hong Kong children learn Chinese through a look-and-say or rote memory approach without the aid of a phonological coding system (Pinyin).

Two follow-up cross-sectional studies of Hong Kong adolescents with dyslexia demonstrated similar findings to those reported in the study of children with dyslexia; that is, adolescents exhibited multiple cognitive-linguistic deficits in rapid naming, visual-orthographic knowledge, morphological skills, and verbal working memory (Chung et al., 2010, 2011). These multiple causes of cognitive-linguistic deficits may reflect poor orthographic and morphological representations and weak linkages among phonological, orthographic, and semantic processors, slower access to the lexicon and activation of phonological representations, and/or weakness in the manipulation and storage of verbal information in working memory, thereby leading to severe reading and writing difficulties (Chung et al., 2010, 2011). Such multiple causes of dyslexia are in line with the multiple deficit model of developmental disorders (Pennington, 2006; Peterson & Pennington, 2012). Based on the multiple deficit model, individuals with dyslexia may suffer from multiple and single underlying causes or risk factors that influence the course and outcome of reading and writing across the lifespan.

Although multiple deficits have been found in Chinese children and adolescents with dyslexia, less attention has been paid to what happens to dyslexic children as they grow into adolescents and whether the cognitive-linguistic profiles of these adolescents may change from childhood through adolescence. A small but growing body of studies on alphabetic languages has investigated adolescent and adult dyslexics who nevertheless have managed to improve their cognitive-linguistic skills such as morphological skills and literacy skills like word reading so that they may be considered as high-functioning dyslexics (e.g., Birch & Chase, 2004; Law et al., 2015). Explanations include the possibility that these individuals have benefitted from the ability to use compensatory strategies and resources such as drawing on their intact morphological skills (Cavalli, Duncan, Elbro, Ahmadi, & Cole, 2017), exploiting their general cognitive abilities (de Beer, Engels, Heerkens, & van der Klink, 2014) and using semantic context, vocabulary knowledge, contextual clues, and world knowledge, or perhaps they have had special training in morphological skills (Elbro & Arnbak, 1996; Nation & Snowling, 1998; Snowling, 2000; Binder & Borecki, 2008; Thompkins & Binder, 2003). Nevertheless, it remains to be further understood how the cognitive-linguistic manifestations of high-functioning dyslexics can possibly be improved by using different degrees of compensation and individual strengths to compensate for literacy difficulties. As yet, little attempt has been made to investigate the characteristics of high-functioning dyslexics and typical-functioning dyslexics from childhood through adolescence.

Given that multiple cognitive-linguistic deficits causing reading and writing difficulties are found in many dyslexic individuals, we explored which ones emerge as stronger and weaker over time in Hong Kong Chinese adolescents with dyslexia. The present study aimed to establish cognitive-linguistic profiles of high-functioning and typical-functioning dyslexics with longitudinal cohorts of Hong Kong Chinese-speaking adolescents diagnosed with childhood dyslexia, allowing us to understand the underlying characteristics of these two dyslexic groups and to determine the permanence of the problem. The two groups of dyslexic students were assessed at Time 1, Grade 2, and reassessed at Time 2, Grade 8, at which stage they were labeled as high-functioning dyslexics and typical-functioning dyslexics. To be labeled as high-functioning dyslexics, students were required to achieve a higher composite score on the word-level skills tests including Chinese Word Reading, One-Minute Reading and Chinese Word Dictation in The Hong Kong Test of Specific Learning Difficulties in Reading and Writing for Junior Secondary School Students (HKT-JS) (Chung et al., 2007). The cutoff score was set at =7 (or 1 standard deviation below the normative mean), where the normative mean and standard deviation were M = 10 and SD = 3. Typical-functioning dyslexics were those who possessed a composite score on the word-level skills of at least 1 standard deviation below the normative mean of their respective ages in the HKT-JS. Similar criteria have been used for English speakers of dyslexia (Kemp, Parrila, & Kirby, 2009; Law et al., 2015).

The present study specifically examined whether high-functioning dyslexics and typical-functioning dyslexics would exhibit different cognitive-linguistic profiles from those of the typical-developing adolescents and, if so, in what way. The performance of both dyslexic groups was therefore compared to the typically developing adolescents of the same chronological age control (CA) in the four cognitive-linguistic constructs, namely visual-orthographic knowledge, morphological skills, rapid naming, verbal working memory, and literacy skills at Time 2. We expected that the two dyslexic groups would show poorer visual-orthographic knowledge, morphological skills, rapid naming, verbal working memory, and literacy skills than the CA group. Relative to the high-functioning dyslexics, we anticipated that typical-functioning dyslexics might show greater difficulties in visual-orthographic knowledge, morphological skills, rapid naming, and verbal working memory based on previous work on dyslexia in Chinese (e.g. Chung et al., 2010, 2011). According to the multiple deficits model, the number of cognitive-linguistic deficits manifested by an individual is linked to the severity of his or her reading and writing problems (e.g. Chung et al., 2011; Ho, Chan, Lee, Tsang, & Luan, 2004; Peterson & Pennington, 2012). Thus, compared to the high-functioning dyslexics, we expected that typical-functioning dyslexics might show more severe impairments in multiple cognitive-linguistic domains that would probably contribute to greater difficulties in reading and writing. Additionally, we expected that rapid naming deficits would be identified in many dyslexic adolescents because previous studies have highlighted rapid naming as a major core cause of dyslexia in Chinese and English (e.g. Bruck, 1992; Chung et al., 2010, 2011; Miller-Shaul, 2005).

## Methods

### Participants

#### Typical-functioning dyslexics

The students for the present study came from a sub-sample of the group of 254 Hong Kong Chinese-speaking students with dyslexia recruited for a longitudinal study. They had been followed from Grade 2 to Grade 8, having been referred by the local education authority and other non-governmental organizations. The sample was representative of three regions: Hong Kong Island, Kowloon, and New Territories in Hong Kong. The typical-functioning students with dyslexia were assessed by professional psychologists in accordance with the diagnostic criteria based on the Hong Kong Test of Specific Learning Difficulties in Reading (HKT-SpLD) (Ho, Chan, Tsang, & Lee, 2000) and an intelligence test from the Hong Kong Wechsler Intelligence Scale for Children (HK-WISC) at Time 1. The HKT-SpLD is a diagnostic test used to assess Hong Kong primary school children with developmental dyslexia. Local norms are available from 6 years 1 month to 10 years 6 months. This assessment battery (HKT-SpLD) includes 12 subtests that are grouped under five domains, namely, literacy skills (Chinese Word Reading, One-Minute Reading, and Chinese Word Dictation), rapid naming, phonological awareness, verbal working memory, and orthographic awareness. Details of these literacy and cognitive-linguistic measures are further discussed in the Assessment section. Raw scores of each subtest were converted to 12 scaled scores according to children’s age levels and the scaled scores ranged from 1 to 19, with 10 being the mean. The literacy composite scores and at least one of the cognitive-linguistic composite scores of the children in the group were at least 1 standard deviation (*SD* = 3) below the normative mean (*M* = 10) of their respective ages in the HKT-SpLD (cutoff score = 7). These 12 subtests have overall reliability coefficients over 0.7. Also, required was a normal intelligence IQ of 85 or above using the Hong Kong Wechsler Intelligence Scale for Children (HK-WISC, Psychological corporation, 1981). These are the diagnostic criteria for the diagnosis of developmental dyslexia in Hong Kong. Furthermore, the students were carefully screened to ensure that they had sufficient learning opportunities (e.g., new immigrants were excluded) and that they did not have any suspected brain damage, uncorrected sensory impairment, serious emotional and behavioral problems. Twenty-five typical-functioning dyslexic students with 5 females and 20 males (mean age = 159.76 months, *SD* = 12.40) from grade 8 were selected to age match with the high-functioning dyslexics group.

#### High-functioning dyslexics

The high-functioning students with dyslexia were also selected from the same sample of the larger dyslexic group as mentioned above. These students had a documented history of dyslexia having been diagnosed with developmental dyslexia at Time 1. However, they had relatively less severe manifestations of reading impairment at Time 2. The grouping criteria of high-functioning dyslexics were based solely on their literacy skills at Time 2. Particularly, their composite scores of Chinese Word Reading, One-Minute Reading, and Chinese Word Dictation in The Hong Kong Test of Specific Learning Difficulties in Reading and Writing for Junior Secondary School Students (HKT- JS) (Chung, Ho, Chan, Tsang, & Lee, 2007) were higher than the cutoff score = 7 (or 1 standard deviation below the normative mean), where the normative mean and standard deviation were *M* = 10 and *SD* = 3 respectively. These literacy composite scores were aligned with the composite scores of literacy skills (Chinese Word Reading, One-Minute Reading, and Chinese Word Dictation) used in identifying students with dyslexia at Time 1. The HKT-JS is a standardized test for diagnosis of developmental dyslexia with norms in Hong Kong. This assessment battery consists of 15 subtests grouped under five domains, namely literacy skills, morphological skills, visual-orthographic knowledge, rapid naming, and verbal working memory. Note that phonological awareness was not included in this test, because a phonological awareness deficit is rarely found in Hong Kong Chinese adolescents with dyslexia (Chung et al., 2010; Ho et al., 2004). Details of these subtests are described in the Assessment section. Fifteen of these subtests have reliability coefficients over 0.7. By this definition, 25 students including 15 male and 10 female (mean age = 159.08 months, *SD* = 14.08) from the original dyslexia group showed such improvement.

#### Typically developing controls

Another 25 typically developing students as chronological age controls (CA) were recruited from two secondary representative schools in Hong Kong at Time 2. Of these, 15 male and 10 female (mean age = 160.00 months, *SD* = 14.24) students were matched by age to the typical-functioning and high-functioning dyslexic students (see Table 1). These students, who were average performers, were nominated by their class teachers based on their previous grade point average for one school year. This grade point average was at the 50–75 percentile in the students’ Chinese language/literature. None of these students had any history of developmental dyslexia or psychopathology in childhood according to teachers’ recommendation. These 25 students were carefully selected to match as closely as possible to the typical-functioning and high-functioning dyslexic groups by age (see Table 1).

**Table 1:** Characteristics of the three groups of participants

	HF (*n* = 25)	TF (*n* = 25)	CA (*n* = 25)	*F* (2, 30)	*p*
M (SD) of:					
IQ	106.87 (9.10)	105.43 (12.59)	111.75 (17.31)	0.45	.64
Age (time1)	107.64 (19.87)	109.20 (19.36)	–	3.08	.06
Age (time2)	159.08 (14.08)	159.76 (12.40)	160.00 (14.24)	1.85	.18
Gender ratio:					
F:M	2:3	1:4	2:3		

HF: high-functioning dyslexics, TF: typical-functioning dyslexics, CA: chronological age controls

### Assessment measures for time 1

At Time 1, each of the five domains of interest, namely literacy, phonological awareness, orthographic awareness, verbal working memory, and rapid naming, was assessed using multiple measures from HKT-SpLD (Ho et al., 2000). The composite score of each domain was calculated by averaging the standardized scores of all relevant measures as outlined below.

#### Literacy

There were three literacy tests: Chinese Word Reading, One-Minute Reading, and Chinese Word Dictation. In the Chinese Word Reading test, students were asked to read aloud 150 Chinese two-character words graded according to levels of difficulty. This test was discontinued when the participants failed to read 15 words consecutively. In the One-Minute Reading test, participants were requested to read aloud 90 two-character words as quickly and accurately as possible within 1 minute. In the Chinese Word Dictation test, participants were asked to write 48 two-character words. Testing stopped if the participants failed to write eight consecutive words correctly.

#### Phonological awareness

The Rhyme Detection and Onset Detection tests consisted of 18 and 15 trials. In each trial of the subtests, students heard three Chinese syllables presented. The Chinese syllables were names of common objects. The participants were asked to indicate which two among the three syllables sounded similar.

#### Orthographic awareness

The Left/Right Reversal, Lexical Decision, and Radical Position tests were used to assess individuals’ knowledge of Chinese character structure. The Left/Right Reversal subtest consisted of 70 simple Chinese integrated characters and Arabic numbers. Half of them were left/right reversed. The participants were asked to cross out all items with an incorrect orientation. In the Lexical Decision test, there were 30 rare characters and 30 non-characters. Radicals of non-characters were placed in illegal positions. The characters were printed in a fixed random order on two pieces of A4 paper. Participants were requested to cross out all non-characters. The Radical Position test consisted of 20 semantic and phonetic radicals. The participants were asked to indicate from the four options (left, right, top, and bottom) the legal position of each radical. The total score in each subtest were calculated by the sum of correct items.

#### Verbal working memory

In tasks of Word Repetition I, Non-Word Repetition, and Word Repetition II, the 15, 14 and 15 trials, were used to test individuals’ verbal working memory. Individual syllables in the two Word Repetition tests were real characters, while those in the Non-word Repetition test were phonetically legal syllables but were non-sense characters in Cantonese. The stimuli were presented to the participants who were asked to repeat the syllables in the presented order.

#### Rapid naming

The Rapid Digit Naming test consisted of 8 rows of 5 digits (2, 4, 6, 7, and 9) that were printed on a white A4 sheet. These digits were arranged in random order. The participants were asked to name all digits on the sheet from left to right and from top to bottom as accurately and quickly as possible. They were asked to name the list twice.

### Assessment measures for time 2

All of the measures for Time 2 were from HKT-JS (Chung et al., 2007). The measures were: literacy, visual-orthographic knowledge, verbal working memory, rapid naming, and morphological skills. The composite score was calculated by averaging the standardized scores of all relevant measures as shown below:

#### Literacy

The Chinese Word Reading, One-Minute Reading, Reading Comprehension, Chinese Word Dictation, and 10-Minute Writing test were used to assess individuals’ reading and writing ability. For the Chinese Word Reading, participants were requested to read 143 Chinese two-character words in order of difficulty. The test was discontinued when students failed to read 20 words consecutively. For the One-Minute Word Reading, participants were asked to read aloud each of the 120 simple two-character words as quickly and accurately as possible within 1 minute. The Reading Comprehension task comprised one narrative, one descriptive, and one expository passage and each passage consisted of six multiple-choice questions. This task had a total of 18 questions. The passages and questions were designed in ascending order of difficulty. For the Chinese Word Dictation test, students were asked to write 47 two-character words. Testing stopped if the participants failed to write eight consecutive words correctly. For the 10-Minute Writing test, participants were given 2 minutes to plan their writing based on a picture depicting a scene in a park. They were then asked to write a description of the scene in the given picture within 10 minutes.

#### Visual-orthographic knowledge

In Character Matching test, there were 18 items. In each item, a target character was presented to the participants and they were asked to select the target character from nine stimuli. The stimuli were composed of a combination of five types of errors: illegal positions, inverted components, one component combining with different components, incorrect number of strokes, and incorrect orientation. For example, the target character  was presented simultaneously with the stimuli that looked like the target character including a component combined with a different component (e.g. , , ), a component contained with missing strokes (e.g. , ), the component was in a mirror orientation (e.g. ), a left/right reversed position (e.g. ), and incorrect positions (e.g. , ).

The Delayed Copying was derived from Pak et al. (2005) study. It consisted of 17 characters in which 14 pseudocharacters and 3 real characters as filler items were presented visually. Of the 14 pseudocharacters, half had 2 component characters (e.g. ) and another half had 3 component characters (e.g. ). Each character was presented to the participants at a rate of 1 character per second and then was removed. As the participants carried out this task, they were required to count backwards from 20 to 11 aloud to prevent them from rehearsing the characters before they reproduced them on paper.

#### Verbal working memory

The Backward Digit Span and Non-word Repetition tasks were used to assess participants’ verbal working memory. For the Backward Digit Span test, there were 14 sequences in lists starting from two digits to a maximum of eight digits. Two sequences were presented at each string length and the list length was increased by one after every two sequences. The students were asked to listen to a series of digits and then required to repeat the list of numbers in the reverse order from that with which they were originally presented. For the Non-word Repetition test, there were 14 trials with three to eight syllables that were presented to participants. Individual syllables were phonetically legal syllables but were monosyllabic non-words in Cantonese. The participants were asked to repeat orally the syllables in the presented order.

#### Rapid naming

Tasks of Rapid Digit Naming and Rapid Letter Naming were included in this test battery. On the Rapid Digit Naming test, participants were presented with an A4 size paper consisting of 10 rows of 5 digits (2, 4, 6, 7, and 9). Participants were asked to read the number names aloud as rapidly and accurately as possible. Similar to the Rapid Digit Naming test, English letters were used in the Rapid Letter naming because these stimuli were familiar to all students. Hong Kong Chinese children learn these alphabet letters in kindergarten. Participants were asked to read a series of lowercase letters (a, u, y, p, t, b, i and o) as quickly as they could for this particular task. The students named all letters presented on the list twice.

#### Morphological skills

For the Morpheme Production task, 14 sentences with a missing morpheme (a character) were presented orally to the participants. Individuals were told to replace the missing word orally. For example, ‘我地要對自己有信 ___’ (We need to have____ in ourselves). One possible correct response was <心 > (heart). The word formed by adding the morpheme <心> was <信心> (confidence).

The Morpheme discrimination task included 17 items, each consisting of four two-character words. In each item, a character that shared the same sound and written form but with a different meaning when combined with the other characters was presented visually and orally to participants. For example, the character <安> /on1/ is the common character in the words <安靜> /on1 zing6/ (quiet), <安排> /on1 paai4/ (arrange), <安全> /on1 cyun 4/ (safe), and <安定> /on1 deng6/ (stable). The participants were then asked to identify the ‘odd’ word. The correct response for this one is <安排> /on1 paai4/ (arrange) because the character <安> /on1/ in the word <安排> /on1 paai4/ represents a different meaning (arrange) from the character <安> /on1/ in the other three words (calm, at ease).

## Results

Of the 50 students diagnosed with dyslexia at Time 1, 25 were identified to be high-functioning dyslexics whereas the other 25 were considered to be typical-functioning dyslexics. The average mean scaled scores on each of the literacy and cognitive-linguistic subtests for the two groups are shown in Table 2. This table reports the means, standard deviations, t-values, and Cohen’s d values for both groups on literacy, phonological awareness, orthographic awareness, verbal working memory, and rapid naming. In the group comparison analyses at Time 1, no significant differences were found in the measures between these two groups of students.

**Table 2:** Mean, standard deviations, *T* test and Cohen’s *d* on various measures for the high-functioning dyslexics and typical-functioning dyslexics at Time 1

	*M (SD)*		*t* (48)	*d*
	HF (*n* = 25)	TF (*n* = 25)		
Age (months)	107.64 (19.87)	109.20 (19.36)	−0.28	−0.08
Literacy:	5.83 (0.90) #	5.65 (1.22) #	0.57	0.16
Chinese word reading	6.44 (1.78) #	6.28 (1.99) #	0.3	0.08
One-minute reading	6.36 (1.98) #	6.24 (1.92) #	0.22	0.06
Chinese word dictation	4.68 (2.06) #	4.44 (1.87) #	0.43	0.12
Phonological awareness:	9.32 (1.83) #	9.08 (2.14) #	0.43	0.12
Rhyme detection	9.00 (2.16) #	9.28 (2.09)	−0.47	−0.13
Onset detection	9.64 (2.55)	8.88 (3.11)	0.94	0.27
Orthographic skills:	8.00 (2.02) #	8.68 (2.29) #	−1.11	−0.31
Lexical decision	7.76 (3.29) #	9.16 (2.93)	−1.59	−0.45
Radical position	8.88 (2.80)	9.28 (2.79)	−0.51	−0.14
Left-right reversal	7.36 (3.50) #	7.60 (3.79) #	−0.23	−0.07
Verbal working memory:	9.35 (2.98) #	9.13 (3.28) #	0.24	0.07
Word repetition I	9.64 (3.29)	9.40 (3.55)	0.25	0.07
Word repetition II	8.60 (3.16) #	8.40 (3.69) #	0.21	0.06
Nonword repetition	9.80 (3.55)	9.60 (3.74)	0.19	0.05
Rapid naming:	5.40 (3.06) #	6.28 (3.36) #	−0.97	−0.27
Digit rapid naming	5.40 (3.06) #	6.28 (3.36) #	−0.97	−0.27

All *p* > .05. HF: high-functioning dyslexics, TF: typical-functioning dyslexics. # Significantly less than the normative mean (10)

At Time 2, the high-functioning dyslexics and typical-functioning dyslexic adolescents were matched with the chronological-age control (CA) group as shown in Table 3. The performances of the all three groups were transformed into standard scores with a mean of 10 and standard deviation of 3. Table 3 shows the means, standard deviations, and F-values from one-way analyses of variance (*ANOVAs*) comparing the three groups on literacy, visual-orthographic knowledge, morphological skills, verbal working memory, and rapid naming. The high-functioning dyslexic group performed significantly better than the typical-functioning group but worse than the CA on literacy, visual-orthographic knowledge, and morphological skills. Both dyslexic groups were performed less well than the CA for rapid naming. The high-functioning dyslexic group performed better than the typical-functioning group on word reading, one-minute reading, reading comprehension, character matching, and morpheme production. Furthermore, the high-functioning dyslexics performed similarly to the control group on the reading comprehension and character matching tasks.

**Table 3:** Means and standard deviations on various measures for the high-functioning dyslexics, typical-functioning dyslexics, chronological-age control group and *f* values for group differences on various tasks at Time 2

Time 2 measures	*M (SD)*			*F*	Post hoc
	HF (*n* = 25)	TF (*n* = 25)	CA (*n* = 25)		
Age (months)	159.08 (14.08)	159.76 (12.40)	160.00 (14.24)	0.03	
Literacy:	7.55 (1.09)	5.86 (1.55)	10.50 (2.08)	52.18***	CA > HF > TF
Chinese word reading	7.08 (2.20)	4.96 (2.78)	11.12 (2.55)	38.54***	CA > HF > TF
One-minute reading	6.60 (2.38)	5.36 (2.10)	9.08 (1.61)	21.27***	CA > HF > TF
Chinese word dictation	4.88 (1.56)	5.00 (2.10)	10.36 (3.50)	38.46***	CA > HF = TF
Ten-minute writing	9.04 (3.01)	8.00 (3.28)	11.64 (3.73)	7.82***	CA > HF = TF
Reading comprehension	10.16 (1.55)	5.96 (3.38)	10.28 (2.79)	20.98***	CA = HF > TF
Visual-Orthographic knowledge	8.56 (2.41)	7.08 (2.04)	10.46 (1.97)	15.50***	CA > HF > TF
Chinese character matching	8.12 (2.67)	6.28 (3.22)	9.24 (2.47)	7.10**	CA = HF > TF
Delayed copying	9.00 (3.64)	7.88 (3.26)	11.68 (2.23)	9.92***	CA > HF = TF
Verbal working memory:	8.84 (2.47)	9.04 (1.90)	9.70 (2.80)	0.87	
Backward digit span	8.20 (3.01)	7.76 (2.54)	9.88 (3.49)	3.39*	CA > TF
Nonword repetition	9.48 (3.39)	10.32 (3.09)	9.52 (3.20)	0.54	
Rapid naming:	5.54 (2.94)	5.24 (2.80)	9.48 (2.47)	18.60***	CA > HF = TF
Digit rapid naming	5.28 (3.16)	5.52 (2.83)	9.04 (2.68)	13.20***	CA > HF = TF
Letter rapid naming	5.80 (3.11)	4.96 (3.08)	9.92 (2.50)	20.84***	CA > HF = TF
Morphological skills:	8.72 (2.07)	7.04 (2.27)	10.18 (1.86)	14.34***	CA > HF > TF
Morpheme discrimination	8.88 (3.18)	7.44 (2.90)	9.80 (3.04)	3.82*	CA > TF
Morpheme production	8.56 (2.62)	6.64 (3.15)	10.56 (1.73)	14.59***	CA > HF > TF

HF: high-functioning dyslexics, TF: typical-functioning dyslexics, CA: chronological age controls,

***p < .001, **p < .01, *p < .05

The four domains of literacy, visual-orthographic knowledge, rapid naming, and verbal working memory were analyzed across Time 1 and Time 2. The time (Time 1 and Time 2) by group (high-functioning dyslexics and typical-functioning dyslexics) repeated measures ANOVAs on the cognitive-linguistic measures indicated significant group-by-time interactions on literacy, *F(1,48)* = 14.53, *p* < .001, and visual-orthographic knowledge, *F(1,48)* = 8.25, *p* < .01, but were non-significant for rapid naming, *F(1,48)* = 3.45, *p* = .074, and verbal working memory, *F(1,48)* = 0.20, *p* = .66. The main effects of group and time in literacy were significant, with *F(1,48)* = 11.18, *p* < .01 and *F(1,48)* = 23.30, *p* < .001 respectively. For the typical-functioning dyslexic group, follow-up contrasts showed a significant decline in visual-orthographic knowledge, *F*(1,24) = 9.43, *p* < .01, and rapid naming, *F*(1,24) = 4.29, *p* < .05, while no significant changes were found in verbal working memory, *F*(1,24) = 0.02, *p* = .89, and literacy, *F*(1,24) = 0.48, *p* = .50. For the high-functioning dyslexic group, significant improvement was found in literacy, *F*(1,24) = 10.14, *p* < .001. But no significant changes were observed for visual-orthographic knowledge, *F*(1,24) = 1.07, *p* = .31, rapid naming, *F*(1,24) = 0.12, *p* = .73, and verbal working memory, *F*(1,24) = 0.68, *p* = .42.

To further evaluate the magnitude of various cognitive-linguistic and literacy measures, the effect sizes of improvement between the two groups were computed. A comparably large effect size was found for visual-orthographic knowledge (*d* = 0.81) and literacy (*d* = 1.08). A medium effect size was found for rapid naming (*d* = 0.52), and a small effect size (*d* = −0.13) was obtained for verbal working memory. These effects indicated that the high-functioning dyslexics had greater improvement in visual-orthographic knowledge, literacy, and rapid naming compared to the typical-functioning dyslexics.

To investigate the heterogeneous profiles of high-functioning dyslexics and typical-functioning dyslexic individuals involved in the present study, the number of deficits across cognitive-linguistic domains was further analyzed. Similar to a number of previous studies (e.g., Birch & Chase, 2004; Chung et al., 2010), this study adopted the criterion of at least 1 standard deviation (or equivalent to a scaled score below 7) below the normative mean as the cutoff criteria for impairment. Table 4 shows the number and percentage of high-functioning dyslexic and typical- functioning dyslexic individuals for each domain and measure at Time 1. Two groups exhibited similar cognitive-linguistic deficits profile. On average, 10%, 29%, 17%, and 56% of the high-functioning dyslexics exhibited deficits in phonological awareness, orthographic skills, verbal working memory, and rapid naming respectively. Similarly, 8%, 24%, 24%, and 48% of the typical-functioning dyslexics displayed deficits in phonological awareness, orthographic skills, verbal working memory, and rapid naming. Two proportions *z*-tests were used to investigate the difference in the proportion of deficits between the two groups. As shown in Table 4, there were no statistically significant differences between the groups with respect to all the cognitive-linguistic domain (all *p* > .5).

**Table 4:** The number of individuals in the high-functioning dyslexics and typical-functioning dyslexics exhibiting deficits in the various cognitive-linguistic areas at Time 1

Measures	HF (*n* = 25)			TF (*n* = 25)		
	*N*	%	Average (%)	*n*	%	Average (%)
Phonological awareness:			10%			8%
Rhyme detection	3	12%		2	8%	
Onset detection	2	8%		2	8%	
Orthographic skills:			29%			24%
Lexical decision	8	32%		4	16%	
Radical position	5	20%		5	20%	
Left-right reversal	9	36%		9	36%	
Verbal working memory:			17%			24%
Word repetition I	3	12%		5	20%	
Word repetition II	6	24%		8	32%	
Nonword repetition	4	16%		5	20%	
Rapid naming:			56%			48%
Digit rapid naming	14	56%		12	48%	

HF: high-functioning dyslexics, TF: Typical-functioning dyslexics

At Time 2 (see Table 5), on average, 24%, 22%, 66%, and 18% of the high-functioning dyslexics exhibited deficits in visual-orthographic knowledge, verbal working memory, rapid naming, and morphological skills respectively. By way of comparison, 46%, 26%, 66%, and 42% of the typical-functioning individuals with dyslexia had deficits in visual-orthographic knowledge, verbal working memory, rapid naming, and morphological skills. It is worth noting that 6%, 20%, 12%, and 8% of the typically developing students had deficits in visual-orthographic knowledge, verbal working memory, rapid naming, and morphological skills, suggesting that a number of students had some difficulty with some cognitive-linguistic skills. Comparing the proportion differences between high-functioning and typical-functioning dyslexics, two proportions z-tests showed that the high-functioning group had a marginally lower proportion of morphological deficits than the typical-functioning dyslexics, *z* = −1.89, *p* = .06, particularly in the morpheme discrimination task, *z* = −2.75, *p* < .01. For visual-orthographic deficits, the high-functioning dyslexics showed a marginally significant lower proportion compared to their typical-functioning dyslexics, *z* = −1.66, *p* = .10. There was also a significant difference in the Chinese character matching task, *z* = −2.05, *p* < .05. However, the difference in rapid naming and verbal working memory between two dyslexic groups was not significant, *z* = 0.00, *p* = 1.0 and *z* = −0.34, *p* = .74 respectively. Comparing the proportion differences between high-functioning dyslexics and typically developing students, both groups showed a similar performance in visual-orthographic knowledge (marginally), *z* = 1.82, *p* = .07, verbal working memory, *z* = 0.18, *p* = .86, and, morphological skills, *z* = 1.07, *p* = .28. However, the high-functioning dyslexics performed significantly worse than the typically developing students in rapid naming, *z* = 3.99, *p* < .001.

**Table 5:** The number  of individuals in the high-functioning dyslexics and typical-functioning dyslexics exhibiting deficits in the various cognitive-linguistic areas at Time 2

Measures	HF (*n* = 25)			TF (*n* = 25)			CA (*n* = 25)		
	*n*	%	Average (%)	*n*	%	Average (%)	*n*	%	Average (%)
Visual-Orthographic knowledge:			24%			46%			6%
Chinese character matching	7	28%		14	56%		3	12%	
Delayed copying	5	20%		9	36%		0	0%	
Verbal working memory:			22%			26%			20%
Backward digit span	7	28%		12	48%		5	20%	
Nonword repetition	4	16%		1	4%		5	20%	
Rapid naming:			66%			66%			12%
Digit rapid naming	17	68%		16	64%		4	16%	
Letter rapid naming	16	64%		17	68%		2	8%	
Morphological skills:			18%			42%			8%
Morpheme discrimination	4	16%		11	44%		4	16%	
Morpheme production	5	20%		10	40%		0	0%	

HF: high-functioning dyslexics, TF: typical-functioning dyslexics, CA: chronological age controls

We further examined the individual cognitive profile at Time 1 and Time 2. Table 6 presents the number of individuals exhibiting deficits in each domain at Time 1. Of the high-functioning dyslexics, fourteen displayed a single deficit. Three individuals had deficits in two domains and two individuals had deficits in three domains. Rapid naming was the most dominant single deficit (40%). The most dominant double deficit was the visual-orthographic knowledge and rapid naming (8%). The deficits profiles of the typical-functioning dyslexics were similar to those of the high-functioning dyslexics. Thirteen of the typical-functioning dyslexics displayed a single deficit. Five individuals had deficits in two domains and one individual had deficits in three domains. As was the case for the high-functioning dyslexics, rapid naming was the most dominant single deficit (32%). Visual-orthographic knowledge and rapid naming were the prominent double deficit (8%).

**Table 6:** Number of individuals in the high-functioning dyslexic and typical-functioning dyslexic group with deficits in each cognitive-linguistic area and their corresponding mean ages, and literacy skills at Time 1

	HF (*n* = 25)					TF (*n* = 25)				
Cognitive deficits	*n* (%)	Age	CWR	OMR	CWD	*n* (%)	Age	CWR	OMR	CWD
No deficit	6 (24%)	105	6.0	6.7	4.8	6 (24%)	121	6.3	6.7	4.0
Single deficit	14 (56%)	108	6.4	6.4	4.9	13 (52%)	100	5.9	6.1	4.8
Phonological awareness (P)	–	–	–	–	–	–	–	–	–	–
Orthographic skills (O)	2 (8%)	89	6.5	7.0	6.5	1 (4%)	132	8.0	6.0	1.0
Verbal working memory (V)	2 (8%)	112	6.0	9.0	3.5	4 (16%)	105	5.8	7.3	5.0
Rapid naming (R)	10 (40%)	111	6.4	5.8	4.8	8 (32%)	94	5.8	5.5	5.1
Double deficits	3 (12%)	123	8.3	6.0	4.0	5 (20%)	124	7.4	6.8	4.0
P + O	–	–	–	–	–	1 (4%)	126	10.0	6.0	7.0
P + R	1 (4%)	121	10.0	3.0	4.0	–	–	–	–	–
O + V	–	–	–	–	–	1 (4%)	124	9.0	12.0	5.0
O + R	2 (8%)	124	7.5	7.5	4.0	2 (8%)	128	5.0	5.5	2.5
V + R	–	–	–	–	–	1 (4%)	116	8.0	5.0	3.0
Triple deficits	2 (8%)	92	5.5	5.5	4.0	1 (4%)	84	5.0	3.0	5.0
P + O + V	1 (4%)	80	5.0	4.0	5.0	–	–	–	–	–
O + V + R	1 (4%)	103	6.0	7.0	3.0	1 (4%)	84	5.0	3.0	5.0

CWR: Chinese word reading, OMR: One-minute reading, CWD: Chinese word dictation, HF: high-functioning dyslexics, TF: typical-functioning dyslexics. The unit of age is in months

Table 7 presents the number of individuals exhibiting deficits in each domain at Time 2. Of the high-functioning dyslexics, seventeen displayed a single deficit. Five individuals had deficits in two domains. Rapid naming was the major single deficit (48%). The double deficits (8%) were found in both visual-orthographic knowledge and rapid naming. None of the high-functioning dyslexics displayed a deficit in three or more domains. Of the typical-functioning dyslexics, eleven dyslexic adolescents showed a single deficit, most frequent being rapid naming (24%). Seven typical-functioning dyslexic adolescents had deficits in two areas. The most dominant double deficits was rapid naming and morphological skills (12%). Six were found to have impairments across three or more domains. The most prominent of these triple deficits were in rapid naming, morphological skills, and visual-orthographic knowledge (12%). For the typically developing students, five adolescents had a single deficit with verbal-working memory being dominant (16%). Only two of them were found to have difficulties across two domains. As shown in Table 7, individuals who had more deficits tended to manifest lower scores across literacy skills than those who had fewer deficits.

**Table 7:** Number of individuals in the high-functioning dyslexic and typical-functioning dyslexic group with deficits in each cognitive-linguistic area and their corresponding mean ages and literacy skills at Time 2

Cognitive deficits	HF (*n* = 25)							TF (*n* = 25)							CA (*n* = 25)						
	*n*	age	CWR	OMR	CWD	RC	TMW	*n*	age	CWR	OMR	CWD	RC	TMW	*n*	age	CWR	OMR	CWD	RC	TMW
No deficit	3	150	5.3	7.7	4.3	10.3	10.3	1	151	1	4	7	10	14	18	162	11.9	9.4	11.2	12.2	10.5
Single deficit	17	161	7	6.2	5.1	10.3	8.9	11	156	6.5	6.2	5.3	6.5	6.9	5	149	8.8	8.0	8.8	10.2	10.4
Visual-Orthographic knowledge (O)	2	152	6	6.5	5.5	10	11	3	152	7	6.7	5.7	8.3	8.7	1	150	10.0	7.0	11.0	10.0	10.0
Verbal-working memory (V)	2	149	6.5	9	6.5	10.5	10	–	–	–	–	–	–		4	149	8.5	8.3	8.3	10.3	10.5
Rapid Naming (R)	12	166	7.2	5.4	4.6	10.4	8.5	6	159	7.7	5.7	4.7	7.2	5.7	–	–	–	–	–	–	–
Morphological awareness (M)	1	149	8	9	7	9	8	2	152	2.5	7	6.5	1.5	8	–	–	–	–	–	–	–
Double deficits	5	158	8.4	7.4	4.6	9.6	8.6	7	165	4.3	6	5	6.4	9	2	170	9.5	8.5	6.5	10.5	8.0
O + R	2	169	10.5	8	5	8.5	8.5	2	159	3.5	7.5	4	9.5	12.5	1	186	10.0	8.0	7.0	11.0	11.0
O + M	–	–	–	–	–	–	–	1	168	7	8	7	6	10	–	–	–	–	–	–	–
V + R	1	151	5	7	4	11	7	–	–	–	–	–	–		–	–	–	–	–	–	–
V + M	1	158	6	8	5	10	12	1	175	4	6	7	7	9	1	154	9.0	9.0	6.0	10.0	5.0
R + M	1	144	10	6	4	10	7	3	165	4	4.3	4.3	4.3	6.3	–	–	–	–	–	–	–
Triple deficits	–	–	–	–	–	–	–	5	163	4	3.8	4.4	4	8	–	–	–	–	–	–	–
O + V + R	–	–	–	–	–	–	–	1	154	5	2	6	7	7	–	–	–	–	–	–	–
O + R + M	–	–	–	–	–	–	–	3	169	4.7	5	4.3	4	8.3	–	–	–	–	–	–	–
V + R + M	–	–	–	–	–	–	–	1	157	1	2	3	1	8	–	–	–	–	–	–	–
Quadruple deficits	–	–	–	–	–	–	–	1	158	1	1	3	3	7	–	–	–	–	–	–	–

CWR: Chinese word reading, OMR: One-minute reading, CWD: Chinese word dictation, RC: Reading comprehension, TMW: Ten-minute writing, HF: high-functioning dyslexics, TF: typical-functioning dyslexics. The unit of age is in months

## Discussion

This longitudinal study examined the profiles of Chinese adolescents identified as typical-functioning and high-functioning dyslexics. These two groups of 50 dyslexic adolescents (with 25 typical-functioning dyslexics) were assessed in both Grade 2 and Grade 8. Another 25 typically developing adolescents were selected as a control group and were assessed at Grade 8 only. All students were administrated measures of cognitive-linguistic skills and literacy skills. Our results showed that both dyslexic groups performed generally less well than the control group on the measures of rapid naming, visual-orthographic knowledge, morphological skills, verbal working memory, reading and writing skills. In particular, visual-orthographic and morphological deficits appeared to be a greater problem for typical-functioning dyslexics than the high-functioning group with 46 vs 24% exhibiting visual-orthographic deficits and 42 vs 18% with morphological deficits. Compared with the high-functioning dyslexics, the typical-functioning group showed a higher incidence of multiple cognitive-linguistic deficit profiles with significant deficits in rapid naming, suggesting that rapid naming is the most prominent and persistent deficit in Chinese adolescents with dyslexia. Our findings also indicated that typical-functioning dyslexics, who had multiple deficits in cognitive-linguistic domains, showed more difficulties with reading and writing than did high-functioning dyslexics. Our results support the multiple deficit model which suggests that dyslexia may consist of a single underlying deficit and multiple impaired skills (Pennington, 2006; Peterson & Pennington, 2012). These findings are further elaborated below.

Approximately 66 and 66% of typical-functioning dyslexics and high-functioning dyslexics in our sample had rapid naming deficits. In particular, deficits in rapid naming were commonly in association with complementary deficits in verbal working memory, morphological skills, and visual-orthographic skills. The dyslexic groups were overall slower at rapid naming of numbers and letters than the control group. In both cases, slow rapid naming performance can be seen as a multifaceted mechanism of weakness in learning sound-symbol arbitrary associations, difficulties in encoding and retrieving phonological or speech codes in memory problems in detecting and integrating visual and orthographic information in memory, and slowness in the speed of access to the lexicon (e.g. Bowers & Newby-Clark, 2002; Georgiou, Protopapas, Papadopoulos, Skaloumbakas, & Parrila, 2010; Liao, Georgiou, & Parrila, 2008). Because the orthography to phonology mapping is relatively arbitrary and considering the vast number of visual-orthographic patterns and units, homographs, and homophones in Chinese, a relationship between rapid naming and reading has been consistently found in children and adolescents with dyslexia (Wang, Georgiou, Das, & Li, 2012) and thus has been used to differentiate Chinese individuals with dyslexia from those without (e.g. Chung et al., 2010, 2014; Shu, McBride-Chang, Wu, & Liu, 2006). These results are consistent with our findings from previous studies (Chung et al., 2010, 2014) showing that rapid naming is the most dominant type of cognitive-linguistic deficit in Chinese children and adolescents with dyslexia. Similar results have been reported for English speakers of dyslexia (Bruck, 1992; Georgiou, Ghazyani, & Parrila, 2018). The current findings further confirm that for the typical-functioning dyslexics and high-functioning dyslexics, rapid naming is the most prominent deficit that persists from childhood through adolescence.

A second domain of difficulty was visual-orthographic knowledge. Around 46% of typical-functioning dyslexics exhibited deficits in visual-orthographic skills that frequently appeared in relation with rapid naming and morphological deficits. The proportion of visual-orthographic deficits in the typical-functioning group was double (46 vs 24%) that of the high-functioning dyslexics and the difference between two proportions was marginally significant. Compared to the high-functioning dyslexics, it seems that for the typical-functioning dyslexics, knowledge of orthographic structure and implicit knowledge of radical positional and functional regularities has not yet been mastered with the result that they may not have developed strong and intact orthographic representations of the radials, characters, and words. Thus, the typical-functioning dyslexics probably have difficulty accessing and processing orthographic representations in their mental lexicon. Visual-orthographic deficits have been considered an underlying cause of dyslexia and are consistently found in studies of dyslexia in Chinese (e.g. Chung, Tong, & McBride-Chang, 2012; Chung et al., 2010; Ho et al., 2004). As found in previous studies in Chinese children and adolescents with dyslexia (e.g. Chung et al., 2010; Ho et al., 2004), visual-orthographic knowledge was also impaired and persisted into adolescence, as did rapid naming.

Apart from visual-orthographic deficits, approximately 42% of typical-functioning dyslexics exhibited morphological deficits that were frequently found in addition to deficits in rapid naming and visual-orthographic skills. The percentage of typical-functioning dyslexics with morphological deficits in our sample was double that of the high-functioning dyslexics (42 vs 18%) perhaps because of difficulties in identifying and discriminating morphemes, manipulating the morphemic structure, and generalizing morpheme meaning. Such difficulties in turn may hinder typical-functioning dyslexics in establishing semantic representations of morphemes and multi-morphemic network thus affecting reading and writing skills. These findings are in line with previous studies (e.g. Chung et al., 2010, 2011; Shu, Peng, & McBride-Chang, 2008) reporting that morphological deficits remain problematic for the typical-functioning dyslexics.

Surprisingly, our findings indicated that the typical-functioning dyslexics, the high-functioning dyslexics, and the controls did not differ in verbal working memory abilities, indicating that working memory deficits may not be a defining feature in distinguishing between different profiles of dyslexia, or adolescents with versus those without dyslexia. These findings are somewhat inconsistent with previous studies identifying deficits in verbal working memory as a major contributor to dyslexia, and reading and writing difficulties, in both English and Chinese (Archibald & Gathercole, 2006; Chik et al., 2012a; Chik et al., 2012b; Sela, Izzetoglu, Izzetoglu, & Onaral, 2012). At this stage, based on this one initial study, it is hard to produce a conclusive explanation for such an inconsistency. One possible explanation is that our controls were recruited through teachers’ nominations and were matched solely by chronological age. Future studies should not only use random sampling to select controls from a pool of potential participants, but also use IQ tests and measures of socioeconomic backgrounds to ensure that the controls and the dyslexics are comparable on important confounding variables. More generally, however, our findings point to the importance of additional work that examines the role in verbal working memory in understanding the etiology of dyslexia in adolescence.

Our profile analysis showed that approximately 56 and 12% of the high-functioning dyslexics had single and double cognitive-linguistic deficits predominantly caused by problems with rapid naming. As in the case of the typical-functioning dyslexic group, nearly 52 and 20% of the single and two cognitive-linguistic deficits were frequently found in conjunction with the rapid naming, visual-orthographic and morphological deficits. It is noteworthy that compared to the high-functioning dyslexics, the typical-functioning group showed a greater tendency toward multiple impairments in one or more cognitive-linguistic domains. However, in most cases, the typical-functioning dyslexics, who had multiple cognitive-linguistic deficits, also showed lower scores in their literacy tasks, thereby suggesting that some typical-functioning dyslexics have difficulty with reading and writing skills. In accordance with previous studies (Chung et al., 2010, 2014), this study found that the number of cognitive-linguistic deficits is linked with varying degrees of literacy difficulties, particularly with reading impairment, and moreover, Chinese adolescents with dyslexia frequently exhibit multiple deficits with rapid naming being the primary cause. Our findings concur with the multiple deficit perspective (Pennington, 2006; Peterson & Pennington, 2012) proposing that multiple pathways to dyslexia are involved in the presence of single and multiple cognitive-linguistic deficits.

The aforementioned results give rise to the question as to why the high-functioning dyslexics performed better in both visual-orthographic knowledge and morphological skills compared to the typical-functioning dyslexics. One can speculate that possibly after years of text exposure and language experience some individuals with dyslexia may be able to improve their visual-orthographic knowledge and morphological skills over time through, for example, using semantic context and contextual clues and employing metacognitive skills to improve their visual-orthographic knowledge and morphological skills (e.g. Binder & Borecki, 2008; Cavalli, et al., 2017; de Beer et al., 2014; Law et al., 2015). Another possible factor may be a result of the fact that orthographic and morphological instructions have been increasingly introduced in the literacy curriculum and intervention programs in many Hong Kong primary schools (Ho et al., 2012; Yeung, Ho, Chan, & Chung, 2013). Correspondingly, some dyslexic individuals may benefit from the explicit and systematic teaching of orthographic structures and rules and word formation knowledge and skills. This, in turn, is likely to facilitate the learning and development of visual-orthographic and morphological skills. Nevertheless, further investigation is needed to fully understand how visual-orthographic knowledge and morphological skills can be developed for some dyslexic individuals and how different manifestations of visual-orthographic knowledge and morphological skills may be acquired by the high-functioning dyslexics but not the typical-functioning dyslexics from childhood through adolescence.

The current study had at least five limitations that warrant further investigations. First, our study was mainly focused on the literacy performance at word-level skills. In order to have a broader view of literacy development in adolescents with and without dyslexia, future studies should also include the measures of text-level skills, for example, sentence reading and reading comprehension in both primary (Time 1) and secondary (Time 2) school grades. Second, given that vocabulary knowledge is highly correlated with word reading and reading comprehension in Chinese (Zhang & Koda, 2018) and English (Binder, Gote, Lee, Bessette, & Vu, 2017), the inclusion of vocabulary knowledge measure is necessary in future research in order to investigate whether vocabulary knowledge may be different among the typical-functioning dyslexics from both high-functioning dyslexics and typically developing adolescents and how it, in turn, affects individuals’ reading and writing skills. Third, future studies may incorporate the measure of morphological skills in Time 1 to provide a better comparison in determining the underlying morphological deficits from childhood to adolescence. Fourth, it is possible that the high-functioning dyslexics, identified in the present study, might have received different forms of intervention, for example, from their parents, who might have hire a private tutor to provide more intensive training, or simply provided relevant simulation through daily interaction, compared to the typical-functioning dyslexics. This could potentially lead to a reduction in the number of deficits perhaps due to the fact that the high-functioning dyslexics had more opportunity for training or instruction or practice to improve an underlying cognitive-linguistic skill. Future research is needed to examine this issue further. Fifth, the typically developing students’ IQ was not measured due to logistic issues. In the results, seven typically developing students were found to have some difficulties mainly in verbal working memory. In future work, it may be worthwhile to include an IQ test such as WISC as well as measures of students’ socioeconomic status and family background information to screen potential adolescents with cognitive and learning difficulties. Finally, the control group of typically developing students was not included in Time 1. It may be useful in the future to embrace a control group in addition to the two dyslexic groups in order to track development of cognitive-linguistic skills and literacy performance in the three groups.

Despite these limitations, however, this study is unique in having followed up on two groups of Chinese students with dyslexia from childhood through adolescence. Our findings show that rapid naming, visual-orthographic knowledge, and morphological skills are important underpinnings of reading and writing in all dyslexics. Although these two dyslexic groups showed multiple cognitive-linguistic deficits with rapid naming being the most prominent deficit, the typical-functioning dyslexics with multiple deficits tended to exhibit more literacy problems than the high-functioning dyslexics. More importantly, even after many years of education, print exposure and access, adolescents with dyslexia continue to be affected by rapid naming deficits. Perhaps deficits in rapid naming, which is an important underlying correlate and possible cause of dyslexia, is universal across languages and writing systems. Indeed, most with dyslexia have this difficulty, for example, in Chinese, English, Italian, and Arabic (e.g., Chung et al., 2010; Georgiou et al., 2018; Layes, Lalonde, & Rebai, 2017; Tobia & Marzocchi, 2014). Together with other studies, this study sheds further light on some important aspects of multiple deficits, with rapid naming being a major causal correlate across subgroups of Chinese adolescents diagnosed with childhood dyslexia.
